# New Delhi Metallo-beta-lactamase-producing Enterobacter Cloacae – A Rare Multidrug Resistance Strain in a Caucasian Woman

**DOI:** 10.7759/cureus.7177

**Published:** 2020-03-04

**Authors:** Suman Siddamreddy, Sreenath Meegada, Vasuki Dandu, Vijayadershan Muppidi, Ramya Bachu

**Affiliations:** 1 Internal Medicine, Baptist Health Medical Center, North Little Rock, USA; 2 Internal Medicine, The University of Texas Health Science Center/Christus Good Shepherd Medical Center, Longview, USA; 3 Neurology, Baptist Health Medical Center, Little Rock, USA; 4 Internal Medicine, Indiana University Health, Indianapolis, USA; 5 Internal Medicine, Baptist Health-University of Arkansas for Medical Sciences, North Little Rock, USA

**Keywords:** ndm-1, superbug, new delhi metallo beta lactamase, multi drug resistant strain, enterobacteriaceae

## Abstract

New Delhi metallo-beta-lactamase (NDM-1) is a novel metallo-beta-lactamase (MBL) gene carried by some Enterobacteriaceae that induces resistance to most of the antibiotics. First described in a Swedish patient hospitalized in India with an infection due to Klebsiella pneumoniae. NDM-1 makes bacteria resistant to a broad range of beta-lactam antibiotics. These include the antibiotics of the carbapenem family, which are a mainstay for the treatment of antibiotic-resistant bacterial infections. Most of these carbapenem resistant Enterobacteriaceae (CRE) are increasingly recognized in hospital settings and post-acute care settings like long-term acute care settings. Percentage of CRE infections is increasing in the United States of America, and invasive infections with CRE carry high mortality rates and limited treatment options. We here present a rare case of elderly Caucasian woman with CRE cellulitis of both legs with no travel history.

## Introduction

Enterobacteriaceae with New Delhi metallo-beta-lactamase (NDM-1) gene are occasionally reported in the United States of America (USA) from time to time. Most of the early cases in the USA were in those patients who received prior medical care in countries like India, Pakistan, and Middle East [1]. But patients with these strains who had not traveled outside the USA started to emerge from 2012 [2]. Surveillance of these strains is really important for early detection of these patients for appropriate treatment and also to prevent further spread. We report a case of 75-year-old Caucasian female who has chronic venous ulcers infected with NDM-1 Enterobacter. The patient was treated with Tigecycline, improved and was discharged home in stable condition.

## Case presentation

A 75-year-old Caucasian female was admitted to the hospital with bilateral cellulitis and non-healing wounds of both lower extremities. Past history was significant for chronic kidney disease, coronary artery disease, diabetes, bio-prosthetic aortic valve replacement and smoking dependence. She had these wounds for two months and used Silvadene and Vaseline to treat them at home. She also used some unknown oral antibiotics initially and they got better. She has not travelled outside the United States. Later she developed fever, chills with worsening pain and redness of the legs, and was admitted to the hospital. On exam, she had large 16 x 6.5 x 1 cm ulcer in left posterior leg and small 0.5 x 0.5 x 1 cm ulcer in right anterior lower leg, with diffuse erythema and warmth of both legs. Peripheral pulses were palpable. Remaining exam was unremarkable. Admission labs were significant for elevated white blood cell count of 11 k/cmm (Reference range: 5-10 k/cmm), and creatinine of 1.06 mg/dl (Reference range: 0.5-1.2 mg/dl). Wound care specialist was consulted and the left leg wound was debrided at bedside and wound cultures were sent out.

The patient was empirically started on intravenous (IV) Vancomycin and Piperacillin/Tazobactam. Tissue culture grew Enterobacter cloacae (Table 1).

**Table 1:** Wound culture and sensitivities

Specimen: Tissue Leg, Left		
Gram stain result: Gram negative rod.		
Culture: Light Growth Enterobacter Cloacae.		
Positive Carbapenemase		
SUSCEPTIBILITY		
Amikacin	<=8 Sensitive	
Aztreonam	<=4 Sensitive	
Cefazolin	>=64 Resistant	
Cefepime	>16 Resistant	
Ceftazidime	>=64 Resistant	
Ceftriaxone	>=64 Resistant	
Ceftazidime/Avibactam	>32 Resistant	
Ciprofloxacin	0.5 Intermediate	
Gentamycin	<=1 Sensitive	
Levofloxacin	1 intermediate	
Meropenem	8 Resistant	
Piperacillin/Tazobactam	>64 Resistant	
Tigecycline	<=1 Sensitive	
Tobramycin	<= Sensitive	
Trimethoprim/Sulfamethoxazole	>=320 Resistant	

Sensitivities revealed that Enterobacter cloacae was resistant to extended spectrum cephalosporins, an elevated carbapenem MIC and having NDM-1 gene (Table 2).

**Table 2:** Enterobacter negative for KPC, positive for NDM-1

Component	
Specimen description = Tissue	
Klebsiella Pneumoniae Carbapenemase (KPC)--Not detected	
New Delhi metallo-beta-lactamase 1--Detected	

Blood cultures remained negative. Enterobacter was sensitive to Amikacin, Aztreonam, Gentamycin, Colistin, Tobramycin and Tigecycline.

Infectious disease specialist was consulted. Antibiotics were changed to IV Tigecycline and she was treated for one week. Colistin and aminoglycosides were not chosen as she has worsening creatinine during that time. The patient’s wound significantly got better and she was discharged home. Later during an outpatient follow-up, her cellulitis got completely resolved but she had some residual venous stasis changes.

## Discussion

Carbapenem-resistant Enterobacteriaceae (CRE) infections are more recognized and reported worldwide because of the emergence and spread of strains producing carbapenemases. Carbapenemases are carbapenem hydrolyzing beta-lactamases that confer resistance to beta lactams as well as carbapenems. Emergence of bacteria with carbapenemases makes the battle of extreme drug resistance in gram negative bacilli much tougher [3]. There are different mechanisms involved in non-susceptibility to carbapenems among Enterobacteriaceae, including porin mutations that decrease carbapenem penetration with production of certain types of beta lactamases [4]. Carbapenem antibiotics have an important role in treating several gram-negative bacteria producing cephalosporinases and extended spectrum beta lactamases [5].

According to Ambler classification system, there are three classes of carbapenemases: Class A, B, and D beta-lactamases. Class A and D enzymes have a serine-based hydrolytic mechanism, and Class B enzymes are metallo beta lactamases with zinc in active site [6]. New Delhi metallo-beta-lactamase (NDM-1) is a type of B beta-lactamase gene that was first identified in Klebsiella pneumoniae in a Swedish patient treated in India in 2009 [7]. Earlier in the United States, Enterobacter isolates with NDM-1 gene were mostly identified in patients who received prior medical care in countries like India and Pakistan [8]. There were few cases reported with more than one type of carbapenemase producing strains from a single patient [6]. There were some cases reported in the USA where patients received prior care in middle eastern countries as well [9]. Initially the cases reported in the USA were from those patients who received prior medical care in other countries but later indigenous cases without previous travel have been reported [10].

Carbapenemase producing bacterial infections can vary from asymptomatic colonization to clinical infections like urinary tract infections, surgical wound infections, bacteremia, respiratory tract infections and abscesses [11-14]. Susceptibility testing should be done on all carbapenemase producing strains. NDM-1 positive strains have other concomitant resistance mechanisms which incur their resistance to other antimicrobials in addition to beta-lactams [15]. Optimal treatment is uncertain as antibiotic options are limited. Consultation with an infectious disease specialist should be made. Some of the regimens that were used in past to treat these infections are: ceftazidime-avibactam, Meropenem-Vaborbactam; Imipenem-Relebactam, Polymyxins, Tigecycline, Eravacycline, and aztreonam [16-19].

Centers for Disease Control and Prevention (CDC) came up with guidelines to control and spread of CRE [20]. Enhanced hygiene, contact precautions (CP), pre-emptive isolation of high-risk patients, health care personnel education, patient and staff cohorting, antibiotic stewardship, inter facility communication are some of the core measures per CDC guidelines. Hospitalized patients should be placed on full CP for the length of hospitalization and also during future hospitalizations. There were no definitive recommendations on duration of contact precautions for future hospitalizations [20].

## Conclusions

In the current antibiotic era with growing resistance to antibiotics, we should be judicious with antibiotic usage. Several carbapenemase gene carrying bacteria are being identified. Prompt infectious disease consultation should be made and multi-drug resistant infections should be treated with appropriate antibiotics in a timely manner to prevent further antibiotic resistance. At the same time, we should limit overuse and misuse of antibiotics and be aggressive with isolation and prevention of the spread of these multi-drug resistance bacteria like the ones with NDM-1 gene. There should be better laboratory methods for CRE screening and novel preventive interventions as well.
